# Ball versus other attachments in mini implant retained overdenture: a systematic review and meta-analysis

**DOI:** 10.1186/s12903-025-05961-z

**Published:** 2025-04-13

**Authors:** Zin Hnin Pwint Aung, Pyae Phyo Win, Thanapat Sastraruji, Pathawee Khongkhunthian

**Affiliations:** 1 Centre of Excellence for Dental Implantology, Faculty of Dentistry, Chiang Mai University, Mueang Chiang Mai 50200, Suthep, 50200 Muang Chiang Mai Thailand; 2 Cho Thar Dental Clinic, North Okkalapa Township, Yangon, Myanmar; 3 Dental Research Centre, Faculty of Dentistry, Chiang Mai University, Suthep, 50200 Mueang Chiang Mai Thailand

**Keywords:** Mini-implants, Overdenture, Denture precision attachment, Marginal bone loss, Systematic review, Meta-analysis

## Abstract

**Objectives:**

Mini implant retained overdentures have been treated in edentulous patients with promising long-term results. However, various attachment systems in this process remain insufficiently investigated. This systematic review and meta-analysis aimed to compare the effects of the ball and other attachments used in mini-implant overdentures. Marginal bone loss, bite force, implant survival rate, prosthetic maintenance, and complications were assessed.

**Materials and methods:**

A systematic search was conducted across PubMed, Cochrane Library, and Scopus databases until 25th February 2025. This systematic review aimed to find studies that compare ball attachments with other attachment systems in mini dental implant (MDI) overdentures. The primary outcome was marginal bone loss, while the secondary outcomes were maximum bite force, implant survival rate, prosthetic maintenance, and complications. The risk of bias was assessed using the Cochrane risk-of-bias tool for RCTs, and a quantitative meta-analysis was performed.

**Results:**

Of the 561 publications, six randomized clinical trials (101 participants, 234 mini-implants) met the inclusion criteria. Risk of bias assessment revealed three studies with a low risk of bias and three studies with some concerns for risk of bias. There was no significant difference in the marginal bone loss between the ball attachments and others (WMD = 0.15, 95% CI -0.50 to 0.81, *p* = 0.65), though ball attachments performed better than telescopic ones (*P* < 0.05) in subgroup analysis. No significant difference in bite force was found (WMD = -5.29, 95% CI -33.46 to 22.87, *p* = 0.71). Two-year survival rates were 90.9% for ball and 97.8% for bar attachments. The ERA^®^ (Extra-Coronal Resilient Attachment) group required five interventions (sore spot adjustments, relining, nylon replacements), while the ball attachment group required only two (denture repair, nylon cap replacement) over the one-year follow-up period.

**Conclusions:**

Within the limitations of the study, it can be concluded that ball, bar, and ERA^®^ attachments yield similar outcomes in marginal bone loss while telescopic attachments show more statistically significant marginal bone loss (*p* < 0.05). The type of attachment does not significantly affect maximum bite force.

**PROSPERO registration number:**

CRD42024610018.

## Introduction

Two-implant retained overdentures are a widely recognized approach for rehabilitating mandibular edentulism, improving function and patient satisfaction [1–3]. Nevertheless, bone resorption after tooth loss often complicates conventional implant (CDI) placement, particularly in elderly or medically compromised individuals [4, 5]. Mini dental implants (MDIs), which are less than 3 mm in diameter, provide a viable alternative for atrophic alveolar ridges [6]. The Glossary of Oral and Maxillofacial Implants (GOMI) defines MDIs as “dental implants made from biocompatible materials comparable to conventional implants but with reduced dimensions” [7]. First introduced in 1994 and later approved by the FDA for both temporary and long-term use, MDIs are now widely applied in prosthodontics [8–10].

One critical factor in assessing MDIs’ success is marginal bone loss (MBL), as it directly impacts implant stability and longevity [11]. Studies have shown that MDI-supported overdentures exhibit favourable MBL outcomes [12, 13], with Aunmeungtong [14] reporting less MBL in MDIs than CDI overdentures. Monitoring MBL is crucial for assessing implant performance and identifying complications such as peri-implantitis and implant failure [15]. Therefore, optimizing MBL outcomes is essential for the long-term success of MDI overdentures.

Beyond MBL, survival rates, bite force, and prosthetic maintenance influence MDIs’ efficacy. While MDIs offer a cost-effective and minimally invasive alternative for edentulous mandibles, their limited fatigue fracture resistance restricts them to removable prostheses [16, 17]. A systematic review by Jawad [18] reported a 95.63% survival rate for MDI overdentures, while Schiegnitz [19] found a similar rate of 94.7% for narrow-diameter implants (NDIs). Additionally, MDI overdentures enhance bite force and chewing efficiency, contributing to greater patient satisfaction [20]. Moreover, long-term success also depends on effective prosthetic maintenance to manage wear and retention loss [21, 22].

Overdenture success is influenced by attachment selection, surgical techniques, and implant positioning [23]. Attachment choice depends on intraoral anatomy, cost, retention needs and patient preferences [24–26]. Attachments are broadly categorized as splinted or non-splinted systems [27]. Splinted systems use bars to connect implants, securing overdenture with clips or other components [28, 29]. Non-splinted systems include balls/O-rings, Locator, ERA^®^ (Extra-Coronal Resilient Attachment), magnets, or crowns [30]. Among them, ball attachments remain popular for their simplicity and benefits in distributing loads on implants [31, 32]. Over time, they have surpassed bars due to cost-effectiveness, minimal prosthetic space requirements, and ease of hygiene maintenance [33, 34]. Biochemically, different attachment systems exhibit varying degrees of resilience, influencing stress distribution and peri-implant bone adaptation, particularly in the critical first year of loading [35–41]. Therefore, understanding whether attachment design impacts MBL in MDIs is essential.

While conventional implant studies suggest that attachment type does not significantly affect peri-implant bone resorption [42, 43], its impact on MDIs remains unclear due to their unique biomechanics. Most systematic reviews on MDIs primarily compare them to standard-diameter implants, focusing on survival rates, bone loss, and patient satisfaction, but often overlook variations in attachment mechanisms. For instance, Lemos’ review evaluated MDIs for complete overdentures but did not investigate differences in attachment designs [44]. Similarly, Borges’ review compared MDIs to standard implants regarding complications and clinical outcomes but did not specifically address attachment variations within MDIs systems [45]. Therefore, this systematic review aims to fill this gap by assessing the influence of ball attachments versus other systems on clinical outcomes, including MBL, bite force, implant survival, prosthetic maintenance, and complications in MDI-supported overdentures.

## Materials and methods

This systematic review was conducted in adherence to the Preferred Reporting Items for Systematic Reviews and Meta-Analyses (PRISMA) guidelines [46, 47]. This review’s protocol was registered in PROSPERO under the registration number CRD42024610018. The objective of this review was to compare the ball attachment with other attachment systems in MDI overdentures. Eligible study designs included randomized controlled trials (RCTs), controlled clinical trials (CCTs) or comparative retrospective or prospective cohort studies with a minimum follow-up period of one year.

Following the Centre for Evidence-Based Medicine guidelines, the PICO framework was used to formulate the research question: *“In edentulous individuals requiring mandibular mini implant-retained overdentures (P)*,* how does the use of ball attachments (I) compare to other attachment systems (C) influence marginal bone loss (MBL)*,* maximum bite force*,* implant survival rate*,* prosthetic maintenance and complications (O)?”*

### Population (P)

Individuals in optimal overall health with an edentulous mandible necessitating the placement of MDI overdentures.

### Interventions (I)

Ball attachments used in MDI overdentures.

### Comparison (C)

Other attachments rather than ball attachments used in MDI overdentures.

### Outcomes (O)

The primary outcome was marginal bone loss.

Maximum bite force, survival rate, prosthetic maintenance and complications were the secondary outcomes.

### Time (T)

Studies with a minimum follow-up period of one year.

### Study design (S)

Randomized controlled trials (RCTs), controlled clinical trials (CCTs) and comparative retrospective or prospective cohort studies.

### Search strategy

A comprehensive literature search was performed using three electronic search platforms: PubMed, Scopus and Cochrane Library. The search was conducted up to February 25th, 2025, to ensure the inclusion of the most recent studies. No restrictions were applied regarding language, publication year, or study type. Both free-text keywords and Medical Subject Headings (MeSH) terms were utilized to maximize the retrieval of relevant studies.

The search technique was designed in accordance with each database and constructed according to the PICO approach as shown in Table 1. Additionally, manual searches were conducted on the reference list of included studies and the relevant systematic reviews related to MDIs for possible additional studies. Moreover, an additional search was implemented in Google Scholar for the analysis of grey literature with the purpose of minimizing publication bias.

Finally, the screening and search terms were performed using a Boolean combination of the following terms; (mini-implant* OR “mini dental implant*” OR “mini implant*”) AND (overdenture* OR “over denture*” OR over-denture*) OR (“complete denture*” OR “full-lower denture*” OR “full lower denture*”) AND (attachment* OR attach* OR “precision attachment*”) OR (ball* OR bar OR magnet* OR splint* OR telescopic OR " double crown*” OR locator* OR equator*) AND (“clinical outcome*”) OR (“marginal bone loss*”)) OR (“maximum bite force*”) OR (“implant survival rate*”) OR (“prosthetic maintenance and complication*”) AND (“Mandible“[Mesh]).

**Table 1:** Construction of search terms according to PICO format

Patient	(mini-implant* OR “mini dental implant*” OR “mini implant*”) AND (overdenture* OR “over denture*” OR over-denture*) OR (“complete denture*” OR “full-lower denture*” OR “full lower denture*”) AND (“Mandible“[Mesh])
Intervention and comparison	(attachment* OR attach* OR “precision attachment*”) OR (ball* OR bar OR magnet* OR splint* OR telescopic OR " double crown*” OR locator* OR equator*)
Outcome	(“clinical outcome*”) OR (“marginal bone loss*”)) OR (“maximum bite force*”)) OR (“implant survival rate*”) OR (“prosthetic maintenance and complication*”)

### Study selection

#### Inclusion criteria

- Randomized controlled trials (RCTs), controlled clinical trials (CCTs), comparative retrospective or prospective investigations, or cohort studies with adult human participants who wear mandibular overdentures retained by MDIs.
- Studies at least a follow-up of 1 year.
- Research that had been written in English.
- Outcomes reporting details about marginal bone loss, survival rates, maximum bite force, prosthetic maintenance and complications with various attachments in a mini dental implant mandibular overdenture.

#### Exclusion criteria

- Research investigated partial removable prostheses supported by implants.
- Studies that specified dental implants over 3 mm in diameter.
- Studies including mini-implant overdenture attachment in the maxillary arch.

At first, a systematic review software application (Rayyan Web) [48] was employed to eliminate duplicate entries. Two investigators (Z.H., P.P.) independently examined the headings and abstracts of every research study gathered through computerized searches. The full text was gathered for papers that satisfied the inclusion criteria or lacked sufficient information upon abstract review for offering a definitive judgment. Two investigators (Z.H., P.P.) independently assessed an in-depth paper obtained from several electronic and alternative search methodologies to ascertain if the studies fulfilled the eligibility criteria. Conflicts were resolved through discussion. A third investigator (P.K.) was consulted when a resolution could not be attained.

### Data extraction

Systematic data extraction from the included reports was conducted, and two assessors (Z.H. and P.P.) independently verified the data. In the event of discrepancies during the extraction process, a third assessor (P.K.) was consulted, and resolution was achieved through consensus discussion. Peri-implant marginal bone loss or change (MBL) was the primary outcome. Secondary outcomes were implant survival rate and maximum bite force, prosthetic maintenance and complications.

The following data were collected from the included full-text studies:

- The first author’s name and the publication year.
- Research design.
- Number and age of individuals taking part.
- Dimension, length and manufacturer of inserted MDIs.
- Kind of attachment.
- Type of loading.
- Radiographic method.
- Duration of follow-ups.
- Outcomes.

### Quality assessment

The risk of bias was evaluated utilizing the Cochrane Risk of Bias Assessment Tool 2 (RoB 2.0) [49]. The initial author (Z.H.) assessed the selected papers, while the second author (P.P.) then examined any divergent viewpoints about those works. The resultant evaluations were classified according to study methodologies as “Low risk,” “Some Concerns”, or “High risk” of bias, as seen in the following domains:

- Bias arising from the randomization process.
- Bias due to deviations from intended interventions.
- Bias due to missing outcome data.
- Bias in the measurement of outcome.
- Bias in the selection of the reported result.

Studies classified as low risk demonstrated a well-defined randomization process, a clearly described intervention with minimal or no deviations, and little to no loss of follow-up. Additionally, they included appropriate, transparent measurement and reporting of outcomes. If any of these criteria were not fully met, the study was considered to have some concerns or a high risk of bias, depending on the level of uncertainty. The RoB 2 Excel tool was used to evaluate the overall risk of bias for each study, incorporating the reviewer’s critical judgment.

### Data analysis

The meta-analysis was conducted by Z.H. and T.S. by utilizing RevMan (version 5.4) [50]. Means and standard deviations (SD) for marginal bone loss (MBL) and maximum bite force (MBF) were extracted from the studies included in the analysis. If a study reported MBL at multiple sites (mesial, distal, buccal, lingual), the overall mean was calculated, and the SD was pooled. When only an average value was provided, it was directly used for data analysis.

#### MBL data analysis

Three studies done by Jofre [54], Ghoneim [58], and Badr [59] measured MBL at multiple implant sites but provided an average MBL value, which was used directly in the analysis. Jofre reported a single averaged value, Ghoneim provided both site-specific and averaged values (with the latter used), and Badr reported only an averaged MBL value.

The fourth study, conducted by Borges & Shoeib [57], measured MBL at four implant sites but did not provide an overall mean or SD. To obtain these values, the mean was calculated by averaging the four site-specific values, and the pooled SD was determined using the pooled standard deviation formula to account for variability across sites.

For consistency with the inclusion criteria of a minimum one-year follow-up, the data at 15 months were taken from the Jofre study [54], while the data at 12 months were extracted from the Ghoneim [57], Borges [58], and Badr [59] studies. This ensured uniformity in the follow-up period across studies.

#### MBF data analysis

For the analysis of maximum bite force (MBF), the means and SD from each study were directly utilized. Data were extracted based on the study’s inclusion criteria, which required a minimum follow-up of one year. Specifically, for Jofre et al. [55], the data at 15 months were selected, as it was the closest follow-up time to one year. For Ghoneim et al. [58], data at 12 months were used. While both studies measured MBF at multiple time points, only the data corresponding to the one-year follow-up were included to align with the study’s inclusion criteria.

#### Heterogeneity and statistical methods

To compare the ball attachment group with other attachment types, weighted mean differences (WMDs) with 95% confidence intervals (CIs) were calculated for these continuous outcomes. The I² index was used to assess heterogeneity, which reflects the percentage of variability in the results due to heterogeneity rather than chance. The following thresholds were used to interpret I² values: 25% for low heterogeneity, 50% for moderate, and 75% for high heterogeneity [51].

The results were derived using either a fixed-effect model or a random-effects model depending on the heterogeneity. A random-effects model was used when the I² value was 50% or higher, or when clinical heterogeneity (e.g., different measurement methods or follow-up durations) was present. A fixed-effects model was applied when no significant statistical or clinical heterogeneity was detected. Specifically, for marginal bone loss (MBL), a random-effects model was selected due to high statistical heterogeneity (I² = 99%), indicating substantial variability among studies. For maximum bite force (MBF), although statistical heterogeneity was low (I² = 23%), a random-effects model was applied due to notable clinical differences in measurement methods and follow-up durations among studies. A *p*-value of < 0.05 was considered statistically significant. Forest plots were created to visually represent the meta-analysis results.

#### Subgroup analysis

Subgroup analysis was performed to explore how different attachment types used in mandibular MDI-retained overdentures might influence the overall effect estimate. This analysis aimed to determine whether specific attachment types contributed to variations in the overall outcomes.

### Certainty of evidence

Utilizing the GRADE system [52], which distinguishes evidence quality as “high,” “moderate,” “low,” or “very low,” the evidence’s degree of certainty was assessed. This assessment is based on a variety of factors, including risk of bias, imprecision, inconsistency, and indirectness. The GRADEpro software was employed to generate a table that summarized the quality of the evidence [53].

## Results

### Search outcomes

**Fig. 1:**
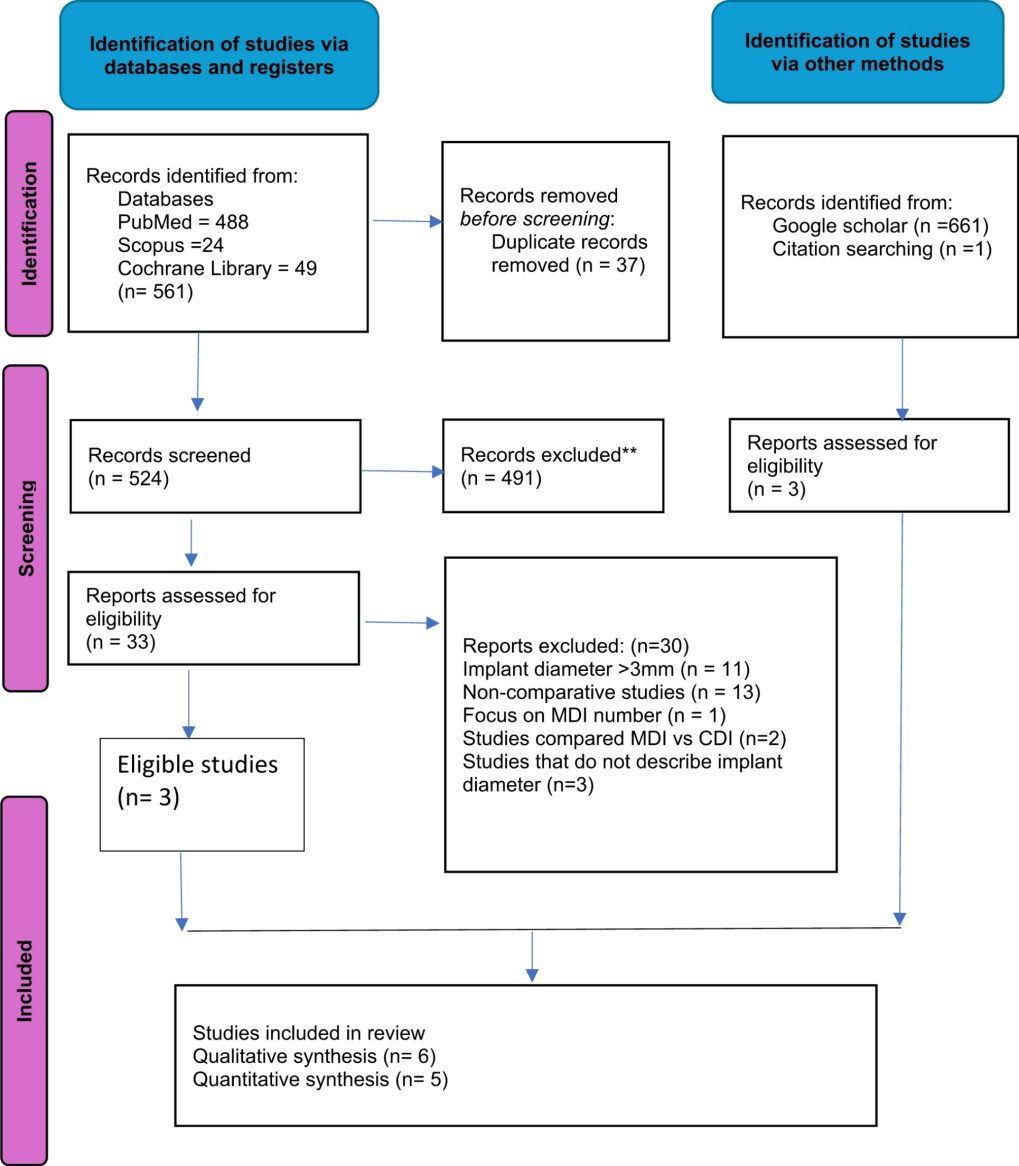
PRISMA 2020 flow diagram illustrating the outcomes of the process

A total of 561 articles were identified through searches across three databases. Duplicate citations were subsequently removed, leaving 524 studies for further evaluation. A thorough assessment of the abstracts led to the exclusion of 491 additional articles, resulting in 33 studies being considered for full-text review. After screening the full texts, three studies met the inclusion criteria, while 30 studies were excluded. Additionally, one study was selected from the reference lists of the included studies, and two studies were identified through Google Scholar. A total of six studies were determined to be appropriate for inclusion, with five studies suitable for quantitative analysis. All the included studies were randomized controlled trials. No eligible controlled clinical trials (CCTs) or comparative cohort studies were identified. The research selection process, conducted in accordance with PRISMA guidelines, was outlined in Fig. 1.

### Study characteristics

Six studies were eligible to be included [54–59] as shown in Table 2. All six included studies were randomized controlled trial designs. The initial three studies were authored by the same individual in the same publication year on the same population; however, all were considered due to their distinct outcomes: the first Jofre’s study [54] addressed marginal bone loss, the second study [55] examined maximum bite force, and the third investigated MDIs survival rate [56]. Therefore, the likelihood of data overlapping may be diminished.

**Table 2:** A brief description of the included MDI studies

Study name & type	Number of patients& age	MDI length, diameter & Company	Follow-up	Radiographic Method	Type of attachment & loading condition	Outcome Parameters MBL (mm) MBF (*N*) SR (%)
(Jofre et al., 2010) [54] RCT	45 NR	Ø1.8 × 15 mm Sendax MDI, IMTEC	5,10,15, and 24 months	Periapical radiograph	Ball & Bar Immediate	**MBL**, At 15 months, Ball = 1.34 ± 1.32 Bar = 0.80 ± 0.58 **MBF** (NR) **SR** (NR)
(Jofre et al., 2010) [55] RCT	45 NR	Ø1.8 × 15 mm Sendax MDI, IMTEC	5,7,10, 15 months		Ball & Bar Immediate	**MBF**, At 15 months Ball = 247.5 ± 139.9 Bar = 203.2 ± 76.8 **SR** (NR)
(Jofre et al., 2010) [56] RCT	45 NR	Ø1.8 × 15 mm Sendax MDI, IMTEC	up to 2 years		Ball & Bar Immediate	**MBL** (NR) **MBF** (NR) **SR**, At 2 years, Group Bar = 97.8% Group Ball = 90.9%
Borg and Shoeib,2021) [57] RCT	20 55–70 years	2.3 mm in diameter Slimline, Dentium, South Korea	6,12 months	CBCT	Ball & Bar Immediate	**MBL**, At 12 months Ball = 0.69 ± 0.32 Bar = 0.85 ± 0.2 **MBF** (NR) **SR (**NR)
(Ghoneim et al.,2021) [58] RCT	20 55–65 years	Ø2.4 × 13 mm NR	12 months	CBCT	Ball & Telescopic Immediate	**MBL**, At 12 months Ball = 0.69 ± 0.05 Telescopic = 1.64 ± 0.09 **MBF**, At 12 months Ball = 184.55 ± 6.53 Telescopic = 184.5 ± 7.33 **SR** (NR)
(Badr,2022) [59] RCT	16 60–65 years	Ø2.4 × 13 mm Cowellmedi Co., Ltd (Korea) Sterngold ERA^®^ (USA)	6,12 months	CBCT	Ball & ERA^®^ Immediate	**MBL**, At 12 months ERA^®^= 0.88 ± 0.24 Ball = 0.92 ± 0.21 **MBF** (NR) **SR** (NR)

Pt’s no = patient’s number, MDI = mini dental implant, MBL = marginal bone loss,

MBF = maximum bite force, SR = survival rate, mm = millimeter, N = newton, NR = not reported,

RCT = randomized controlled trial, CBCT = Cone-beam computed tomography

All studies included follow-up durations minimum of 1 year; two studies [54, 56] had follow-ups of 2 years, one study lasted 15 months [55], and three investigations [57–59] extended up to 12 months. All experiments indicated immediate loading in each case regarding the loading methodology. Concerning the MDIs number, a total of 234 MDIs were distributed to 101 individuals. All studies employed two mini dental implants (MDIs) for the retention of mandibular overdentures, except for one study [59] that utilized four MDIs for denture retention. Four research studies [54, 57–59] provided data on the primary outcome of MBL, two studies [55, 58] offered results for maximum bite force, one study [56] presented findings on MDI survival rate and one [59] evaluated prosthetic maintenance and complications.

### Quality assessment

**Fig. 2:**
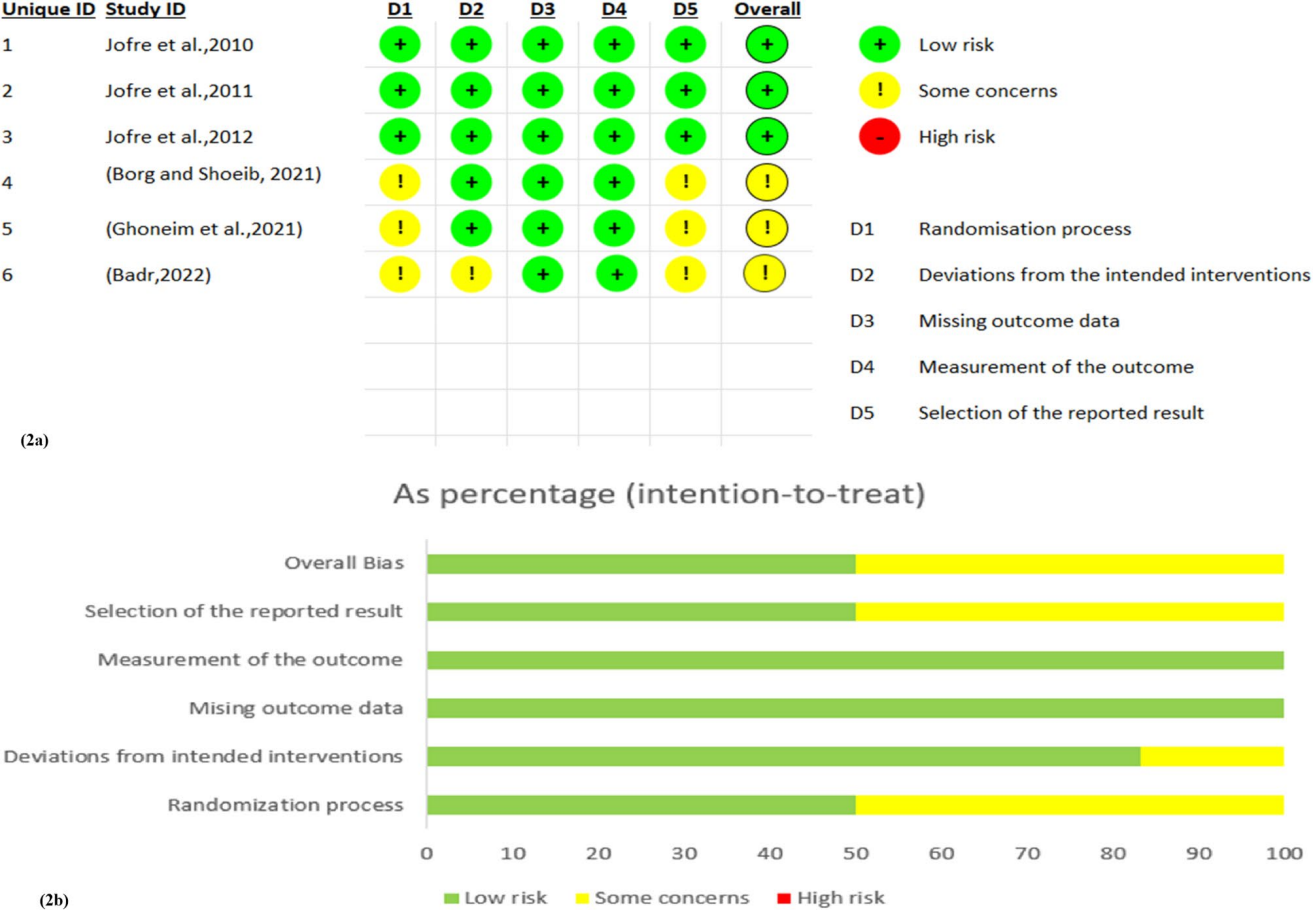
Summary of risk of bias (**2a**) Evaluations of each bias risk criterion for every included study. (**2b**) Representation of risk of bias as a percentage for each of the included studies in a graphical format

Comprehensive explanations of the risk of bias in the included trials are presented in the “Risk of bias” figures, illustrated in Fig. 2a and b. Of the six selected studies, three exhibited some issues in their results [57–59], whilst three were assessed to have a low risk of bias [54–56]. Due to the experimental character of the studies, it was difficult to blind participants and personnel.

The assessment of bias arising from the randomization process indicates low risk in three studies by Jofre [54–56], as they were conducted with independent allocation and random numbers, with no involvement from the surgeon or prosthodontist in patient assignment. In contrast, the studies by Borg [57] and Ghoneim [58] lacked a clear explanation of their randomization methods. In Badr’s study [59], although software was used for randomization, it did not provide adequate details for a thorough risk evaluation. Regarding bias due to deviations from intended interventions, all studies were considered low risk, as they adhered closely to treatment protocols without noted deviations. The risk associated with missing outcome data was also low across all studies. Three studies [54–56] maintained clear documentation of participant randomization and reported dropouts that occurred in one group while retaining overall clarity on participant follow-up. The other studies [57–59] reported no missing outcome data. In the domain of outcome measurement, all studies were classified as low risk, offering clear descriptions of their measurement methods and demonstrating no significant discrepancies between intervention groups. Lastly, concerning bias in the selection of reported results, three studies [54–56] exhibited low risk by registering their protocols and outcomes on ClinicalTrials.gov, allowing for external verification. Conversely, the remaining studies [57–59] raised uncertainties about reporting bias due to the absence of pre-trial protocols, potentially affecting the comprehensiveness and reliability of reported outcomes.

### Qualitative synthesis

Regarding marginal bone loss (MBL), four studies assessed changes in bone levels over time using different imaging techniques. Borg & Shoeib, Ghoneim et al., and Badr et al., [57–59] utilized CBCT (Cone-beam computed tomography) to evaluate crestal bone loss at four implant surfaces (distal, mesial, buccal, and lingual). Measurements were recorded at baseline, six, and twelve months, using standardized reference points. Borges & Shoeib [57] reported the highest bone loss at the buccal site and the lowest at the lingual site, with average values of 0.69 ± 0.32 mm for the ball group and 0.85 ± 0.2 mm for the bar group after 12 months. Similarly, Ghoneim et al., [58] documented 0.69 ± 0.05 mm for the ball group and 1.64 ± 0.09 mm for the telescopic crown group after 12 months. Badr et al., [59] reported bone loss values of 0.88 ± 0.24 mm for the ERA group and 0.92 ± 0.21 mm for the ball group in 12 months. In contrast, Jofre et al., [54] measured MBL using standardized periapical radiographs and a digital caliper, assessing bone levels from the first implant thread to bone contact at mesial and distal sites with follow-ups at baseline, five, ten, fifteen, and twenty-four months. At fifteen months, the mean MBL was 1.34 ± 1.32 mm for the ball group and 0.80 ± 0.58 mm for the bar group. Additionally, Jofre et al., [54] classified 51% of MDIs as exhibiting vertical bone loss and 49% as horizontal bone loss, providing further insights into bone loss morphology.

Maximum bite force (MBF) was evaluated in two studies (Jofre & Hamada and Ghoneim [55, 58]). In the Jofre study [55], MBF was recorded using a thin (98 μm) press-sensitive Dental Prescale sheet at baseline (pre-surgical) and at 5, 7, 10, and 15 months. A progressive increase in MBF was observed in both groups over time, with values reaching 247.53 ± 132.91 N for the ball group and 203.23 ± 76.85 N for the bar group at 15 months. Similarly, Ghoneim [58] measured MBF at the molar and premolar regions on both sides using a portable occlusal force gauge. Measurements were taken at baseline, six, and twelve months. A significant increase in MBF was noted across all follow-up periods, with the highest values recorded at 12 months: 184.55 ± 6.53 N for the ball group and 184.50 ± 7.33 N for the telescopic crown group.

Regarding the implant survival rate, Jofre study [56] compared the survival rates of implants in the ball and bar groups over a two-year follow-up period. After two years, one implant failure was recorded in the ball group (1/46), while four implants failed in the bar group (4/44). The corresponding survival rates were 97.8% for the ball group and 90.9% for the bar group.

Prosthetic maintenance and complications were assessed by Badr et al., [59] who compared ball and ERA attachments through monthly follow-ups. The total number of interventions was five in the ERA group and two in the ball group. In the ERA group, interventions included two sore spot adjustments, one relining, and two nylon male replacements. In the ball group, maintenance involved one denture base fracture repair and one nylon cap replacement (Table 2).

### Quantitative synthesis/meta-analysis

#### Marginal bone loss

**Fig. 3:**
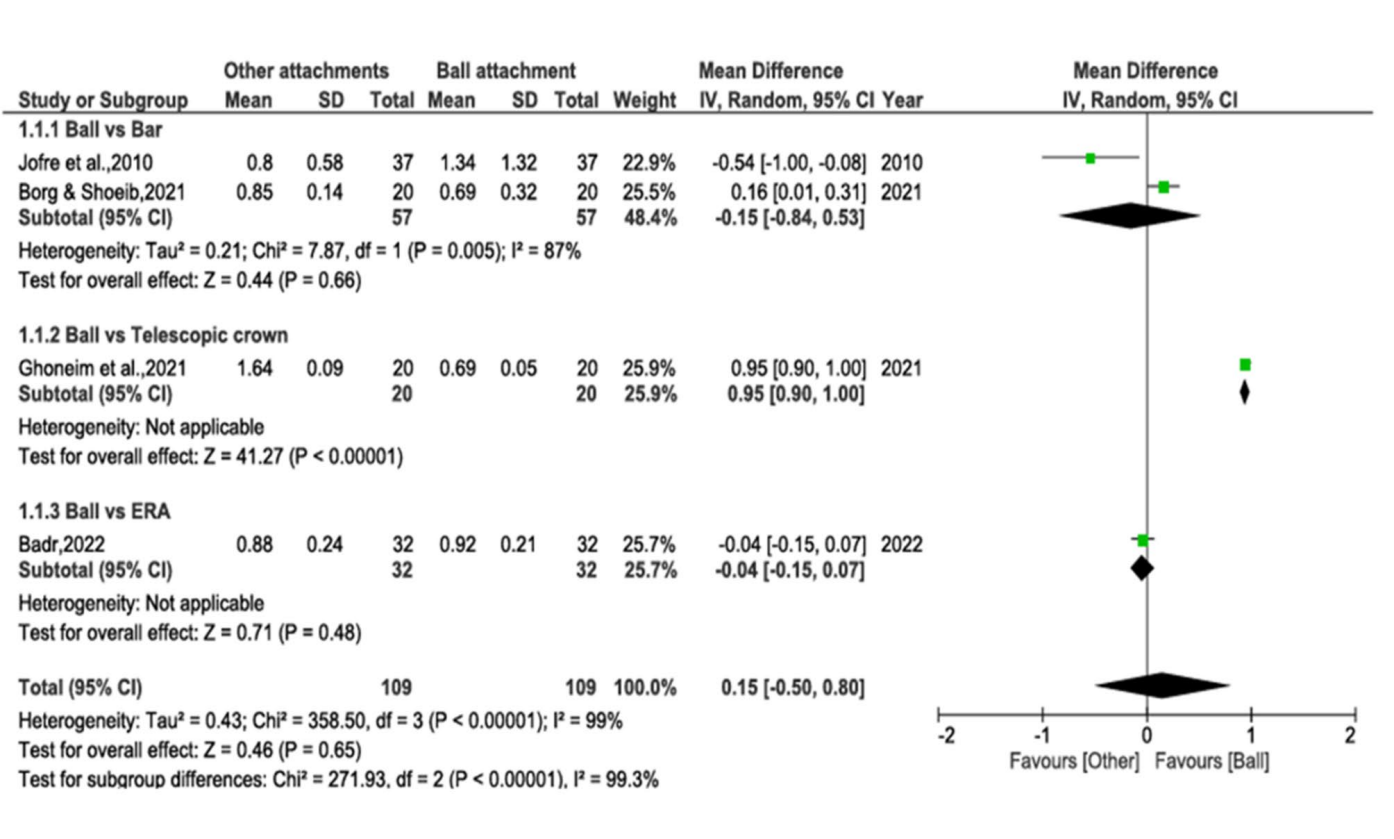
Analysis 1– Comparative assessment of marginal bone loss between ball and bar, ball and telescopic crown, ball and ERA^®^ attachment

The following meta-analysis includes four studies [54, 57–59] with a follow-up of not less than 12 months, comparing the marginal bone loss between ball attachments and other attachment types. The comparison of MBL between the ball and other attachment groups yielded a substantial heterogeneity Chi² value of 350.13 (*P* < 0.00001) and an I² value of 99%. Therefore, instead of the fixed-effect model, DerSimonian–Laird model as a random-effect model was applied. No statistically significant difference of MBL was detected between ball and other attachments (WMD = 0.15, 95% CI– 0.50 to 0.81, *p* = 0.65). Given the non-significant overall effect, the results suggest that there is no clear preference for ball attachments over other types of attachments based on the included studies. (Fig. 3)

#### Marginal bone loss- subgroup analysis

According to the different attachments applied in these studies, three subgroups (i.e. ball vs. bar, ball vs. telescopic crown and ball vs. ERA) showed the comparison of average MBL in the subgroups, respectively. In the ball vs. bar attachments subgroup analysis [54, 57], no significant difference was detected between ball and bar attachments. (WMD − 0.15, 95%CI − 0.84 to 0.53, *p* = 0.66) (Fig. 3).

In ball vs. telescopic attachments [58], a significant difference exists between the two groups, as evidenced by the observation of a statistical significance level of (*P* < 0.05). After analysing the data from this single experiment on bone loss, it was found that the ball attachment had less bone loss compared to the telescopic attachment (WMD = 0.95, 95% CI 0.90 to 1.00, *p* < 0.00001) (heterogeneity = not applicable, total MDIs = 40, one study, Z-score = 41.27).

In ball vs. ERA^®^ attachment [59], both groups did not show a significant difference (*P* > 0.05) when the data from this one experiment was combined (WMD = -0.04, 95%CI -0.15 to 0.07, *p* = 0.48) (heterogeneity = not applicable, total MDIs = 64, one study, Z-score = 0.71).

#### Maximum bite force

**Fig. 4:**
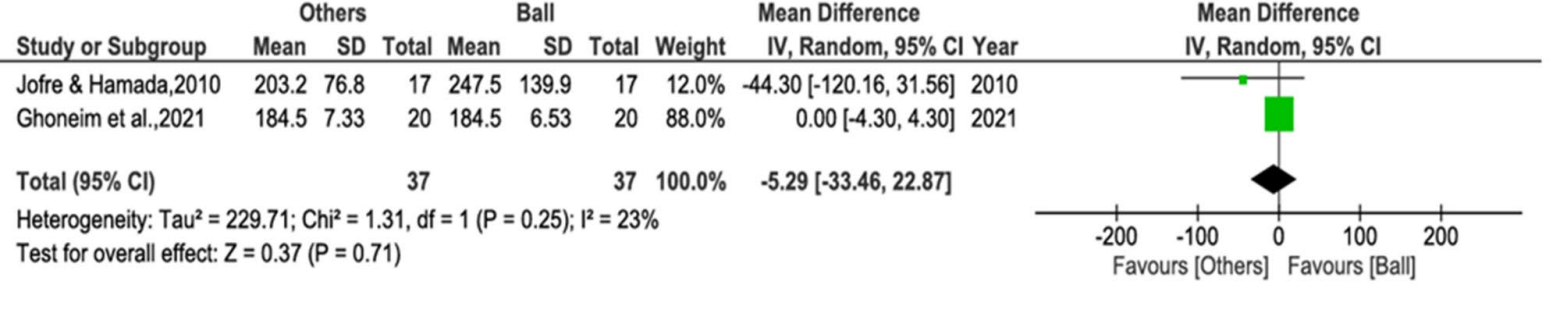
Analysis 2– Comparison of maximum bite force between ball and alternative attachments

The following forest plot includes two studies [55, 58] that measure the maximum bite force of ball attachments versus other attachment types. The heterogeneity among the studies is low, as indicated by a Chi² value of 1.31 with 1 degree of freedom (*P* = 0.25) and an I² value of 23%. It indicates that there is no significant difference in maximum bite force among ball and other attachment types (WMD = -5.29, 95% CI -33.46 to 22.87, *p* = 0.71) (Fig. 4).

### Certainty of evidence

The level of evidence from the marginal bone was judged to be very low, while the maximum bite force is moderate. The level of evidence for marginal bone loss is low due to several limitations. Risk of bias is a concern, as three out of four studies had unclear randomization. The.

evidence was downgraded as very serious for inconsistency (I² = 99%) and serious for imprecision, as the wide CI (WMD = 0.15, 95% CI − 0.49 to 0.80, *p* = 0.64) suggests substantial uncertainty. The certainty is rated as very low, and results should be interpreted with caution. For maximum bite force, the evidence is based on two randomised trials. Serious imprecision was noted due to the wide CI (CI = -33.46 to 22.87), indicating uncertainty. With low heterogeneity (I² = 23%), the certainty is rated as moderate, and results should be interpreted with caution. More details on the level of evidence, based on the GRADE criteria, are described in Table 3.

**Table 3:** Evaluation of the certainty of evidence following GRADE guidelines

Certainty assessment							№ of patients		Effect		Certainty	Importance
№ of study	Study design	Risk of bias	Inconsistency	Indirectness	Imprecision	Other considerations	Ball	others	Relative (95% CI)	Absolute (95% CI)		
**Marginal Bone Loss**												
4	RCT	serious^a^	very serious^b^	not serious	serious^c^	none	109	109	-	MD **0.15 higher** (0.49 lower to 0.8 higher)	⨁◯◯◯ Very low^a, b,c^	CRITICAL
**Maximum Bite force**												
2	RCT	not serious	not serious	not serious	serious^d^	none	37	37	-	MD **5.29 lower** (33.46 lower to 22.87 higher)	⨁⨁⨁◯ Moderate^d^	CRITICAL

**Note: bold values are the exact same number taken from the forest plots (all figures before)**

**CI**: confidence interval; **MD**: mean difference; **RCT**: randomized controlled trial

**Explanations**

^a^. Three out of four studies showed potential risk of bias.

^b^. Heterogeneity (I² = 99%) in marginal bone loss assessment of four studies.

^c^. Wide range of confidence intervals.

^d^. Wide range of confidence intervals and small sample size

## Discussion

This systematic review and meta-analysis compared clinical outcomes of ball attachments versus other attachments in MDI-retained overdentures, evaluating MBL, maximum bite force (MBF), survival rates, prosthetic maintenance, and complications. Six randomized controlled trials with a minimum follow-up period of one year were included in the qualitative analysis, while five were eligible for quantitative analysis.

The meta-analysis revealed no significant difference in MBL between the ball and other attachment types (bar, ERA^®^, telescopic crowns) in MDI overdentures (*P* > 0.05). In subgroup analysis, no significant difference in MBL was found between ball vs. bar or ball vs. ERA (*P* > 0.05), while MBL was significantly lower with ball compared to telescopic crowns (*P* < 0.05). Nevertheless, the average MBL across all attachment groups was under 2 mm, indicating clinically acceptable levels of bone loss [60].

Included 2 studies in this systematic review comparing ball and bar attachments yielded conflicting results. Jofre [54] reported higher MBL in the ball group, whereas Borg and Shoeib [57] found greater MBL in the bar group. This discrepancy may be due to implant diameter, follow-up duration, and bone density. Study implants (MDIs) differed greatly in diameter. Jofre et al. [54] used narrower Ø1.8 × 15 mm Sendax MDI (IMTEC) implants, while Borg and Shoeib [57] used 2.3 mm Slimline (Dentium, South Korea) implants. As narrower implants are subjected to increased biomechanical pressure, which can increase bone resorption [61], this likely contributed to the differences in MBL across the studies. Follow-up duration also varied, with Jofre’s study spanning 15 months and Borg’s 12 months. While both used immediate loading, differences in observation periods could influence reported bone loss. Additionally, neither study provided details on bone density, a crucial factor affecting implant stability and MBL [62]. Variations in bone quality could have contributed to the observed differences in outcomes. Despite these differences, the MBL variation was not statistically significant, supporting evidence that attachment type may not significantly impact MBL. Further research with standardized parameters is needed to confirm these findings.

The certainty of evidence for MBL in this review was rated very low due to a significant risk of bias, high heterogeneity (I² = 99%), and small sample sizes. Consequently, these findings must be interpreted with caution. The uncertainty of MBL is influenced by multiple factors, including implant number, distribution, and design. Among the included studies, only Badr et al. [59] used four MDIs to support overdentures, while Jofre et al. [54], Borg & Shoeib [57] and Ghoneim et al. [58] used two MDIs. This variation in implant number may act as a confounding factor in MBL assessment. Chatrattanarak et al. [63] reported that overdentures supported by two MDIs exhibited less bone loss than those with four MDIs, suggesting that implant distribution influences peri-implant bone remodelling. Similarly, implant design may also contribute to MBL variability. Although all four included studies [54, 57–59] used one-piece MDIs, the implants came from different manufacturers, introducing potential design-related differences that may affect bone response. In the literature, the impact of implant configuration remains unclear. Aunmeungtong et al. [64] found similar stress distributions between one-piece and two-piece MDIs, while Trang et al. [65] observed lower stress levels in one-piece implants, suggesting a potential biomechanical advantage. Therefore, implant design may act as an additional confounding factor in interpreting MBL outcomes. These findings highlight the multifactorial nature of MBL and the need for individualized treatment planning based on patient-specific biomechanical and anatomical considerations.

Regarding maximum bite force (MBF), the certainty of evidence was rated as moderate. The wide confidence interval (CI = -33.46 to 22.87) reflects substantial uncertainty. Although heterogeneity was low (I² = 23%), additional research is required to validate these findings. In statistical analysis, Ghoneim et al. [58] and Jofre & Hamada [55] reported no significant differences in maximum bite force (MBF) between ball and bar or ball and telescopic crown attachments. Both studies observed increased MBF over time as patients adapted to their prostheses; however, methodological differences in MBF measurement (e.g., Dental Prescale sheets in the Jofre study vs. hydraulic pressure gauges in the Ghoneim study) limit the reliability and generalizability of findings.

Regarding survival rates, MDIs demonstrated high success, which is consistent with the findings of Balaji’s study [66], reporting a survival rate of 94.2%. Among the included studies, only one directly compared attachment types, reporting survival rates of 90.9% for ball attachments and 97.8% for bar attachments. Therefore, limited comparative data necessitate further research to assess the impact of different attachment types on survival rates.

When selecting an attachment system, prosthetic maintenance and complications are important considerations. Rosa’s study [67] reported that ball attachment requires less maintenance compared to bar attachments. Similarly, the included study [59] in this review found that ball attachments required fewer interventions than the ERA^®^ attachment, with the ball primarily needing denture repairs and nylon cap replacements, whereas the ERA^®^ group required more frequent adjustments and nylon replacements. These findings suggest that ball attachments may offer advantages in terms of easier maintenance.

Only a few studies met the inclusion criteria for this systematic review. In addition, comparisons were limited to ball vs. bar, ball vs. telescopic crowns, and ball vs. ERA^®^ attachments in MDI overdentures due to the scarcity of available data. Research on other attachment types such as locator, equator, magnetic systems, were mostly case reports or non-controlled studies or comparisons with standard diameter implants using the same attachment type, making them ineligible for inclusion. Small sample sizes in some trials also contributed to the variability in results, as they may lack sufficient statistical power. In addition, heterogeneity in implant diameter, design, length, number, and follow-up duration, along with confounding factors such as patient demographics, occlusal forces, implant positioning, and oral hygiene, may have influenced variability in MBL outcome. Consequently, further well-designed studies with larger sample sizes and longer follow-up periods are needed to validate these findings.

Despite the confounding factors in the analysed studies, MDIs remain a valuable option in prosthodontic rehabilitation, particularly for patients with anatomical, financial, or medical limitations [66, 68]. Moreover, the flapless surgical technique reduces morbidity and recovery time, making MDIs accessible to medically compromised or economically disadvantaged patients [56, 68–74]. Additionally, the immediate loading capability of MDIs allows for same-day prosthodontic rehabilitation, improving patient satisfaction and quality of life [56, 66, 68–72]. While long-term data is still limited, current evidence suggests MDIs achieve satisfactory survival rates.

Within its limitations, this systematic review found no clear superiority among ball, bar, and ERA^®^ attachments in terms of MBL, though telescopic attachments tended to cause more bone loss. However, due to the low certainty of evidence, these findings should be interpreted with caution. No significant differences were observed in maximum bite force between ball, bar, and telescopic crown attachments in MDI overdentures. Given the moderate certainty of evidence, ball attachments may be a suitable choice for MBF. Clinicians should consider factors beyond MBL and MBF, such as patient preferences, ease of maintenance, cost, and oral hygiene when selecting an attachment system for MDI-retained overdentures. Additional long-term research with larger sample sizes is necessary to confirm these findings and further inform clinical decision-making.

## Conclusions

Within the limitations of the study, it can be concluded that ball, bar, and ERA^®^ attachments yield similar outcomes in marginal bone loss while telescopic attachments show more statistically significant marginal bone loss (*p* < 0.05). The type of attachment does not significantly affect maximum bite force.

## Data Availability

We declared that the datasets used and analysed in the manuscript, including all relevant raw data, are available from the corresponding author upon reasonable request.

## Abbreviations

MDIs: Mini dental implants
NDIs: Narrow diameter implants
CDIs: Conventional dental implants
MBL: Marginal bone loss
MBF: Maximum bite force
SR: Survival rate
CBCT: Cone-beam computed tomography
RCTs: Randomized controlled trials
CCTs: Controlled clinical trials
WMD: Weighted mean difference
SD: Standard deviation
CI: Confidence interval
ERA®: Extra-coronal resilient attachment
GOMI: Glossary of oral and maxillofacial implants
FDA: Food and Drug Administration
PRISMA: Preferred reporting items for systematic reviews and meta-analysis
MeSH: Medical Subject Headings
RoB: Risk of bias

## References

[1] Thomason JM, Feine J, Exley C, et al. Mandibular two implant-supported overdentures as the first choice standard of care for edentulous patients—the York consensus statement. Br Dent J. 2009;207(4):185–6. 10.1038/sj.bdj.2009.728. 19696851 10.1038/sj.bdj.2009.728

[2] Feine JS, Carlsson GE, Awad MA, et al. The McGill consensus statement on overdentures: mandibular two-implant overdentures as first choice standard of care for edentulous patients. Int J Oral Maxillofac Implants. 2002;17(4):601–2. 12182304

[3] Meijer HJ, Raghoebar GM, Batenburg RH, Visser A, Vissink A. Mandibular overdentures supported by two or four endosseous implants: a 10-year clinical trial. Clin Oral Implants Res. 2009;20(7):722–8. 10.1111/j.1600-0501.2009.01710.x. 19489933 10.1111/j.1600-0501.2009.01710.x

[4] Visser A, Stellingsma C, Raghoebar GM, Meijer HJ, Vissink A. A 15-year comparative prospective study of surgical and prosthetic care and aftercare of overdenture treatment in the atrophied mandible: augmentation versus nonaugmentation. Clin Implant Dent Relat Res. 2016;18(6):1218–26. 10.1111/cid.12386. 26676082 10.1111/cid.12386

[5] Mundt T, Schwahn C, Stark T, Biffar R. Clinical response of edentulous people treated with mini dental implants in nine dental practices. Gerodontology. 2015;32(3):179–87. 10.1111/ger.12066. 23859086 10.1111/ger.12066

[6] Jung RE, Al-Nawas B, Araujo M, et al. Group 1 ITI consensus report: the influence of implant length and design and medications on clinical and patient-reported outcomes. Clin Oral Implants Res. 2018;29(Suppl 16):69–77. 10.1111/clr.13342. 30328189 10.1111/clr.13342

[7] Laney WR. Glossary of oral and maxillofacial implants. Int J Oral Maxillofac Implants. 2017;32(4). 10.11607/jomi.2017.4.gomi. 10.11607/jomi.2017.4.gomi 28708903

[8] Barber HD, Seckinger RJ. The role of the small-diameter dental implant: a preliminary report on the miniplant system. Compend Contin Educ Dent. 1994;15(11):1390–2. 7758026

[9] Sendax VI. Mini-implants as adjuncts for transitional prostheses. Dent Implantol Update. 1996;7(2):12–5. 9525163

[10] Balkin BE, Steflik DE, Naval F. Mini-dental implant insertion with the auto-advance technique for ongoing applications. J Oral Implantol. 2001;27(1):32–7. 10.1563/1548-1336(2001)027<0032:MIIWTA>2.3.CO;2. 11326539

[11] Serrano Zamora R, Iglesias Velázquez Ó, Gao BX, Martín Carreras-Presas C, López-Quiles J. Immediate versus conventional loading (IL vs. CL) influence on implant marginal bone loss (MBL): a narrative review. Front Oral Maxillofac Med. 2021. 10.21037/fomm-21-51.

[12] Schenk N, Bukvic H, Schimmel M, Abou-Ayash S, Enkling N. One-piece mini dental implant-retained mandibular overdentures: 10-year clinical and radiological outcomes of a non-comparative longitudinal observational study. J Funct Biomater. 2024;15(4):99. 10.3390/jfb15040099. 38667556 10.3390/jfb15040099 PMC11051283

[13] Curado TF, Silva JR, Nascimento LN, Leles JL, McKenna G, Schimmel M, et al. Implant survival/success and peri-implant outcomes of titanium-zirconium mini implants for mandibular overdentures: results from a 1-year randomized clinical trial. Clin Oral Implants Res. 2023;34(8):769–82. 10.1111/clr.14102. 37254798 10.1111/clr.14102

[14] Aunmeungtong W, Kumchai T, Strietzel FP, Reichart PA, Khongkhunthian P. Comparative clinical study of conventional dental implants and mini dental implants for mandibular overdentures: a randomized clinical trial. Clin Implant Dent Relat Res. 2017;19(2):328–40. 10.1111/cid.12461. 27804205 10.1111/cid.12461

[15] Di Domênico MB, Farias Collares K, Bergoli CD, dos Santos MBF, Corazza PH, Özcan M. Factors related to early marginal bone loss in dental implants—a multicentre observational clinical study. Appl Sci. 2021;11(23):11197. 10.3390/app112311197.

[16] Cutbirth ST. Immediate mini implant placement following extractions. Dent Today. 2014;33(100):102, 104–5. 24791304

[17] Tomasi C, Idmyr BO, Wennström JL. Patient satisfaction with mini implant stabilized full dentures: a 1-year prospective study. J Oral Rehabil. 2013;40(7):526–34. 10.1111/joor.12053. 23551029 10.1111/joor.12053

[18] Jawad S, Clarke PT. Survival of mini dental implants used to retain mandibular complete overdentures: systematic review. Int J Oral Maxillofac Implants. 2019;34(2):343–56. 10.11607/jomi.6991. 30883617 10.11607/jomi.6991

[19] Schiegnitz E, Al-Nawas B. Narrow-diameter implants: a systematic review and meta-analysis. Clin Oral Implants Res. 2018;29(Suppl 16):21–40. 10.1111/clr.13272. 30328192 10.1111/clr.13272

[20] Rosa L, Bataglion C, Siéssere S, et al. Bite force and masticatory efficiency in individuals with different oral rehabilitations. Open J Stomatol. 2012;2(1):21–6. 10.4236/ojst.2012.21004.

[21] Mifsud DP, Cortes ARG, Zarb MJ, Attard NJ. Maintenance and risk factors for fractures of overdentures using immediately loaded conventional diameter or mini implants with locator abutments: a cohort study. Clin Implant Dent Relat Res. 2020;22(6):706–12. 10.1111/cid.12952. 33094529 10.1111/cid.12952

[22] van der Moolen PL, Post BJ, Slot DE, van der Weijden FA. Outcome of peri-implant maintenance care in patients with an implant-supported lower denture—a 3.5-year retrospective analysis. Clin Implant Dent Relat Res. 2021;23(2):236–43. 10.1111/cid.12963. 33463040 10.1111/cid.12963 PMC8247953

[23] Flanagan D, Mascolo A. The mini dental implant in fixed and removable prosthetics: a review. J Oral Implantol. 2011;37(Suppl 1):123–32. 10.1563/AAID-JOI-D-10-00052.1. 20553165 10.1563/AAID-JOI-D-10-00052.1

[24] Chaware SH, Thakkar ST. A systematic review and meta-analysis of the attachments used in implant-supported overdentures. J Indian Prosthodont Soc. 2020;20(3):255–68. 10.4103/jips.jips_368_19. 33223695 10.4103/jips.jips_368_19 PMC7654206

[25] Kaddah A. Principles of removable complete prosthodontics: advanced clinical course. Egypt Dent Online Community; 2008.

[26] Shafie H. Principles of attachment selection. Clinical and laboratory manual of implant overdentures. Ames, Iowa: Blackwell; 2007.

[27] Elsyad MA, Khirallah AS. Circumferential bone loss around splinted and nonsplinted immediately loaded implants retaining mandibular overdentures: a randomized controlled clinical trial using cone beam computed tomography. J Prosthet Dent. 2016;116(5):741–8. 10.1016/j.prosdent.2016.03.005. 27174405 10.1016/j.prosdent.2016.03.005

[28] Trakas T, Michalakis K, Kang K, Hirayama H. Attachment systems for implant retained overdentures: a literature review. Implant Dent. 2006;15(1):24–34. 10.1097/01.id.0000202419.21665.36. 16569958 10.1097/01.id.0000202419.21665.36

[29] de Souza Batista VE, de Souza Batista FR, Vechiato-Filho AJ, Lemos CA, Pellizzer EP, Verri FR. Rehabilitation with mandibular implant-retained complete overdenture using the association of two retention systems. J Craniofac Surg. 2016;27(6):e620–2. 10.1097/SCS.0000000000002954. 27513785 10.1097/SCS.0000000000002954

[30] Seo YH, Bae EB, Kim JW, et al. Clinical evaluation of mandibular implant overdentures via locator implant attachment and locator bar attachment. J Adv Prosthodont. 2016;8(4):313–32. 10.4047/jap.2016.8.4.313. 27555901 10.4047/jap.2016.8.4.313 PMC4993845

[31] Preiskel HW. Overdentures made easy: a guide to implant and root-supported prostheses. London: Quintessence Publishing; 1996.

[32] Alsabeeha NH, Payne AG, Swain MV. Attachment systems for mandibular two-implant overdentures: a review of in vitro investigations on retention and wear features. Int J Prosthodont. 2009;22(5):429–40. 20095190

[33] Sadowsky SJ. Treatment considerations for maxillary implant overdentures: a systematic review. J Prosthet Dent. 2007;97(6):340–8. 10.1016/S0022-3913(07)60022-5. 17618916 10.1016/S0022-3913(07)60022-5

[34] Branchi R, Vangi D, Virga A, Guertin G, Fazi G. Resistance to wear of four matrices with ball attachments for implant overdentures: a fatigue study. J Prosthodont. 2010;19(8):614–9. 10.1111/j.1532-849X.2010.00613.x. 20546492 10.1111/j.1532-849X.2010.00613.x

[35] Fromentin O, Picard B, Tavernier B. In vitro study of the retention and mechanical fatigue behavior of four implant overdenture stud-type attachments. Pract Periodontics Aesthet Dent. 1999;11(3):391–7. 10379298

[36] Besimo CE, Guarneri A. In vitro retention force changes of prefabricated attachments for overdentures. J Oral Rehabil. 2003;30(7):671–8. 10.1046/j.1365-2842.2003.01140.x. 12791150 10.1046/j.1365-2842.2003.01140.x

[37] Mericske-Stern R, Assal P, Buergin W. Simultaneous force measurements in three dimensions on oral endosseous implants in vitro and in vivo: a methodological study. Clin Oral Implants Res. 1996;7(4):378–86. 10.1034/j.1600-0501.1996.070412.x. 9151606 10.1034/j.1600-0501.1996.070412.x

[38] Menicucci G, Lorenzetti M, Pera P, Preti G. Mandibular implant-retained overdenture: a clinical trial of two anchorage systems. Int J Oral Maxillofac Implants. 1998;13(6):851–6. 9857597

[39] Mericske-Stern R. Three-dimensional force measurements with mandibular overdentures connected to implants by ball-shaped retentive anchors: a clinical study. Int J Oral Maxillofac Implants. 1998;13(1):36–43. 9509778

[40] Duyck J, Van Oosterwyck H, Vander Sloten J, De Cooman M, Puers R, Naert I. In vivo forces on oral implants supporting a mandibular overdenture: the influence of attachment system. Clin Oral Investig. 1999;3(4):201–7. 10.1007/s007840050102. 10803135 10.1007/s007840050102

[41] Menicucci G, Ceruti P, Barabino E, Screti A, Bignardi C, Preti G. A preliminary in vivo trial of load transfer in mandibular implant-retained overdentures anchored in two different ways: allowing and counteracting free rotation. Int J Prosthodont. 2006;19(6):574–6. 17165296

[42] van Kampen F, Cune M, van der Bilt A, Bosman F. The effect of maximum bite force on marginal bone loss in mandibular overdenture treatment: an in vivo study. Clin Oral Implants Res. 2005;16(5):587–93. 10.1111/j.1600-0501.2005.01121.x. 16164466 10.1111/j.1600-0501.2005.01121.x

[43] Naert I, Gizani S, Vuylsteke M, van Steenberghe D. A 5-year randomized clinical trial on the influence of splinted and unsplinted oral implants in the mandibular overdenture therapy. Part I: Peri-implant outcome. Clin Oral Implants Res. 1998;9(3):170–7. 10.1034/j.1600-0501.1998.090304.x. 10530131 10.1034/j.1600-0501.1998.090304.x

[44] Lemos CA, Verri FR, Batista VE, Júnior JF, Mello CC, Pellizzer EP. Complete overdentures retained by mini implants: a systematic review. J Dent. 2017;57:4–13. 10.1016/j.jdent.2016.11.009. 27888049 10.1016/j.jdent.2016.11.009

[45] Borges GA, Codello DJ, Del Rio Silva L, Dini C, Barão VAR, Mesquita MF. Factors and clinical outcomes for standard and mini-implants retaining mandibular overdentures: a systematic review and meta-analysis. J Prosthet Dent. 2023;130(5):677–89. 10.1016/j.prosdent.2021.11.010. 35120735 10.1016/j.prosdent.2021.11.010

[46] Fleming PS, Seehra J, Polychronopoulou A, Fedorowicz Z, Pandis N. A PRISMA assessment of the reporting quality of systematic reviews in orthodontics. Angle Orthod. 2013;83(1):158–63. 10.2319/032612-251.1. 22720835 10.2319/032612-251.1 PMC8805538

[47] Moher D, Liberati A, Tetzlaff J, Altman DG, PRISMA Group. Preferred reporting items for systematic reviews and meta-analyses: the PRISMA statement. PLoS Med. 2009;6(7):e1000097. 10.1371/journal.pmed.1000097. 19621072 10.1371/journal.pmed.1000097 PMC2707599

[48] Ouzzani M, Hammady H, Fedorowicz Z, Elmagarmid A. Rayyan—a web and mobile app for systematic reviews. Syst Rev. 2016;5:210. 10.1186/s13643-016-0384-4. 27919275 10.1186/s13643-016-0384-4 PMC5139140

[49] Sterne JA, Savović J, Page MJ, et al. RoB 2: a revised tool for assessing risk of bias in randomised trials. BMJ. 2019;366:l4898. 10.1136/bmj.l4898. 31462531 10.1136/bmj.l4898

[50] Cochrane Collaboration. Review manager (RevMan) [Computer program]. Version 5.4. Copenhagen: The Nordic Cochrane Centre, The Cochrane Collaboration; 2014.

[51] Higgins JP, Thompson SG, Deeks JJ, Altman DG. Measuring inconsistency in meta-analyses. BMJ. 2003;327(7414):557–60. 10.1136/bmj.327.7414.557. 12958120 10.1136/bmj.327.7414.557 PMC192859

[52] Guyatt GH, Oxman AD, Vist GE, Kunz R, Falck-Ytter Y, Alonso-Coello P, et al. GRADE: an emerging consensus on rating quality of evidence and strength of recommendations. BMJ. 2008;336:924–6. 10.1136/bmj.39489.470347.AD. 18436948 10.1136/bmj.39489.470347.AD PMC2335261

[53] GRADEpro GDT, GRADEpro GDT. GRADEpro guideline development tool. McMaster University and Evidence Prime; 2021. Available from: gradepro.org.

[54] Jofre J, Cendoya P, Munoz P. Effect of splinting mini-implants on marginal bone loss: a Biomechanical model and clinical randomized study with mandibular overdentures. Int J Oral Maxillofac Implants. 2010;25(6):1137–44. 21197490

[55] Jofré J, Hamada T, Nishimura M, Klattenhoff C. The effect of maximum bite force on marginal bone loss of mini-implants supporting a mandibular overdenture: a randomized controlled trial. Clin Oral Implants Res. 2010;21(2):243–9. 10.1111/j.1600-0501.2009.01834.x. 20070758 10.1111/j.1600-0501.2009.01834.x

[56] Jofré J, Conrady Y, Carrasco C. Survival of splinted mini-implants after contamination with stainless steel. Int J Oral Maxillofac Implants. 2010;25(2):351–6. 20369095

[57] Borg H, Shoeib A. Comparison of marginal bone loss in mandibular overdentures supported by two solitary versus splinted mini-implants. Egypt J Oral Maxillofac Surg. 2021;12(1):70–6. 10.21608/omx.2021.84074.1125.

[58] Ghoneim WM, Badawy MM, Alhadad DF. Comparison of stud and telescopic crown attachments for mini-implant retained mandibular complete overdentures. Al-Azhar J Dent Sci. 2021;24(4):369–77. 10.21608/ajdsm.2020.47104.1124.

[59] Badr AMI. Effect of using two different mini dental implant attachments on marginal bone height and prosthetic maintenance in implant retained mandibular overdenture. Ain Shams Dent J. 2022;26(2):6–14. 10.21608/asdj.2022.160486.1139.

[60] Misch CE, Perel ML, Wang HL, Sammartino G, Galindo-Moreno P, Trisi P et al. Implant success, survival, and failure: The International Congress of Oral Implantologists (ICOI) Pisa Consensus Conference. Implant Dent. 2008;17(1):5–15. 10.1097/ID.0b013e3181676059 10.1097/ID.0b013e3181676059 18332753

[61] Santinoni CD, Batista VE, Oliveira HF, Lemos CA, Cruz RS, Verri FR. Biomechanical analysis of narrow dental implants for maxillary anterior rehabilitation. Rev Odontol UNESP. 2023;52:e20230027. 10.1590/1807-2577.02723.

[62] Merheb J, Vercruyssen M, Coucke W, Quirynen M. Relationship of implant stability and bone density derived from computerized tomography images. Clin Implant Dent Relat Res. 2018;20(1):50–7. 10.1111/cid.12579. 29277972 10.1111/cid.12579

[63] Chatrattanarak W, Aunmeungtong W, Khongkhunthian P. Comparative clinical study of conventional dental implant and mini dental implant-retained mandibular overdenture: A 5- to 8-year prospective clinical outcomes in a previous randomized clinical trial. Clin Implant Dent Relat Res. 2022;24(4):475–87. 10.1111/cid.13098. 35675561 10.1111/cid.13098

[64] Aunmeungtong W, Khongkhunthian P, Rungsiyakull P. Stress and strain distribution in three different mini dental implant designs used in implant-retained overdentures: A finite element analysis study. Oral Implantol (Rome). 2016;9(4):202–12. 10.11138/orl/2016.9.4.202. 28042449 10.11138/orl/2016.9.4.202 PMC5159942

[65] Trang BNH, Kanazawa M, Murakami N, et al. Stress distribution of one-piece and two-piece mini-implant overdentures with various attachment systems and diameters: A finite element analysis. J Prosthodont Res. 2023;67(3):430–6. 10.2186/jpr.JPR_D_22_00108. 36372437 10.2186/jpr.JPR_D_22_00108

[66] Balaji A, Mohamed JB, Kathiresan R. A pilot study of mini implants as a treatment option for prosthetic rehabilitation of ridges with sub-optimal bone volume. J Maxillofac Oral Surg. 2010;9(4):334–8. 10.1007/s12663-010-0152-2. 22190820 10.1007/s12663-010-0152-2 PMC3177481

[67] Rosa CDDRD, de Souza Leão R, Guerra CMF, Pellizzer EP, Silva Casado BGD, Moraes SLD. Do ball-type attachment systems for overdenture result in better patient satisfaction? A systematic review of randomized crossover clinical trials. Saudi Dent J. 2021;33(6):299–307. 10.1016/j.sdentj.2021.03.002. 34434031 10.1016/j.sdentj.2021.03.002 PMC8376671

[68] Griffitts TM, Collins CP, Collins PC. Mini dental implants: an adjunct for retention, stability, and comfort for the edentulous patient. Oral Surg Oral Med Oral Pathol Oral Radiol Endod. 2005;100:e81–4. 10.1016/j.tripleo.2005.06.018. 16243233 10.1016/j.tripleo.2005.06.018

[69] Elsyad MA, Gebreel AA, Fouad MM, Elshoukouki AH. The clinical and radiographic outcome of immediately loaded mini implants supporting a mandibular overdenture: A 3-year prospective study. J Oral Rehabil. 2011;38(11):827–34. 10.1111/j.1365-2842.2011.02213.x. 21972846 10.1111/j.1365-2842.2011.02213.x

[70] Cho SC, Froum S, Tai CH, Cho YS, Elian N, Tarnow DP. Immediate loading of narrow diameter implants with overdentures in severely atrophic mandibles. Pract Proced Aesthet Dent. 2007;19(3):167–74. 17511121

[71] Shatkin TE, Shatkin S, Oppenheimer BD, Oppenheimer AJ. Mini dental implants for long-term fixed and removable prosthetics: A retrospective analysis of 2514 implants placed over a five-year period. Compend Contin Educ Dent. 2007;28(2):92–9. 17319180

[72] Mazor Z, Steigmann M, Leshem R, Peleg M. Mini-implants to reconstruct missing teeth in severe ridge deficiency and small interdental space: A 5-year case series. Implant Dent. 2004;13(4):336–41. 10.1097/01.id.0000148554.83439.00. 15591995 10.1097/01.id.0000148554.83439.00

[73] Ahn MR, An KM, Choi JH, Sohn DS. Immediate loading with mini dental implants in the fully edentulous mandible. Implant Dent. 2004;13(4):367–72. 10.1097/01.id.0000148560.65514.3d. 15591999 10.1097/01.id.0000148560.65514.3d

[74] Douglass CW, Shih A, Ostry L. Will there be a need for complete dentures in the united States in 2020? J Prosthet Dent. 2002;87(1):5–8. 10.1067/mpr.2002.121203. 11807476 10.1067/mpr.2002.121203

